# Hexavalent chromium–induced epigenetic instability and transposon activation lead to phenotypic variations and tumors in *Drosophila*

**DOI:** 10.1093/eep/dvac030

**Published:** 2022-12-28

**Authors:** Rasesh Y Parikh, Vamsi K Gangaraju

**Affiliations:** Department of Biochemistry and Molecular Biology, Medical University of South Carolina, 173 Ashley Avenue, Charleston, SC 29425, USA; Hollings Cancer Center, Medical University of South Carolina, Charleston, SC 29425, USA; Department of Biochemistry and Molecular Biology, Medical University of South Carolina, 173 Ashley Avenue, Charleston, SC 29425, USA; Hollings Cancer Center, Medical University of South Carolina, Charleston, SC 29425, USA

**Keywords:** hexavalent chromium, epigenetic instability, tumor, developmental robustness, canalization, epigenetic inheritance

## Abstract

Developmental robustness represents the ability of an organism to resist phenotypic variations despite environmental insults and inherent genetic variations. Derailment of developmental robustness leads to phenotypic variations that can get fixed in a population for many generations. Environmental pollution is a significant worldwide problem with detrimental consequences of human development. Understanding the genetic basis for how pollutants affect human development is critical for developing interventional therapies. Here, we report that environmental stress induced by hexavalent chromium, Cr(VI), a potent industrial pollutant, compromises developmental robustness, leading to phenotypic variations in the progeny. These phenotypic variations arise due to epigenetic instability and transposon activation in the somatic tissues of the progeny rather than novel genetic mutations and can be reduced by increasing the dosage of Piwi - a Piwi-interacting RNA–binding protein, in the ovary of the exposed mother. Significantly, the derailment of developmental robustness by Cr(VI) exposure leads to tumors in the progeny, and the predisposition to develop tumors is fixed in the population for at least three generations. Thus, we show for the first time that environmental pollution can derail developmental robustness and predispose the progeny of the exposed population to develop phenotypic variations and tumors.

## Introduction

Developmental robustness (also known as canalization) refers to the ability of an organism to resist phenotypic variations despite inherent genetic variations and environmental perturbations. Developmental robustness was initially conceptualized by Conrad Waddington [1], but molecular insights into the same remained elusive until the discovery of the role of heat shock protein 90 (Hsp90) in developmental robustness [2, 3]. The mutation or pharmacological inhibition of Hsp90 led to the expression of cryptic genotype variations, which are usually silenced. This argued for a mechanism in which Hsp90 acts as a capacitor for morphological evolution, where the expression of cryptic variations allows rapid evolutionary changes in response to environmental pressure/stress. Furthermore, using a genetic screen, an epigenetic mechanism was proposed by which the Trithorax-group proteins function along with Hsp90 in silencing cryptic variations [4]. More recent work has shown that such an epigenetic mechanism can involve the regulation of the influence of endogenous retroviruses on neighboring genes [5].

*Drosophila* Hsp83 (Hsp90), co-chaperone Stip1, and Piwi - a Piwi-interacting RNA (piRNA)-binding protein, interact with each other and promote developmental robustness [6]. This work led to the hypothesis that in times of stress, Hsp83 and Piwi functions would be compromised to increase genetic and epigenetic variability, leading to phenotypic variations that can then be subjected to natural selection and fixed in a population in subsequent generations [7]. Here, we test if environmental stress induced by industrial pollutants derails developmental robustness, leading to transgenerational effects. Environmental pollution is a significant worldwide problem with detrimental consequences for human development. Specifically, the effect of exposure to environmental pollutants on subsequent generations remains a considerable void in our understanding of the long-term effects of pollution.

**Figure 1:**
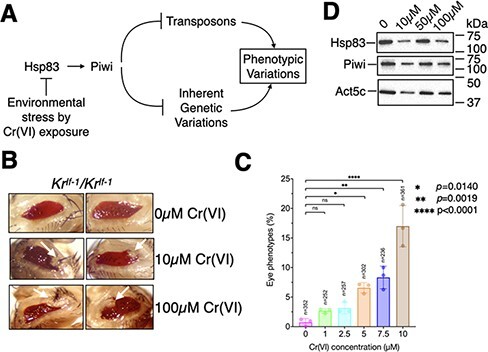
Cr(VI) exposure leads to developmental defects in the progeny. (a) A flowchart representing our working hypothesis. (b) Eye developmental defects (white arrows) observed in the progeny of flies exposed to various concentrations of Cr(VI). (c) Quantitation of developmental defects. ‘*n*’ indicates the number of flies scored for developmental defects. (d) Immunoblot results showing the levels of indicated proteins in various Cr(VI)-exposure conditions

In this work, we used hexavalent chromium, Cr(VI), a potent industrial pollutant, as the stressor since Cr(VI) has been shown to inhibit Hsp83 expression in human cell lines [8]. We reasoned that Cr(VI) exposure–induced inhibition of the Hsp83 function would derail developmental robustness and lead to phenotypic variations in the progeny (Fig. 1a). The US Environmental Protection Agency, the International Agency for Research on Cancer, and the National Institute of Occupational Safety and Health all classify Cr(VI) compounds as human carcinogens. Several million workers worldwide and about half a million in the USA face occupational exposure to Cr(VI) compounds. Occupational exposure mainly occurs via chronic inhalation and dermal contact with manufacturing products. Environmental exposure happens by the discharge of Cr(VI) contaminants in the drinking water supply chain, affecting millions of people, and by the release into the air via burning fossil fuel. An example of Cr(VI) pollution includes groundwater contamination in the town of Hinkley, CA, where residents are still concerned about the presence Cr(VI) in well water.

Much of the genotoxicity by Cr(VI) compounds is induced when Cr(VI) is reduced to Cr(III) intracellularly [9–11]; genotoxic events include DNA damage via DNA strand breaks [12–15], DNA interstrand crosslinks [16, 17], and DNA oxidation [18–23]. Cr(VI) does not directly interact with DNA. However, intermediate products of Cr(VI) reduction, Cr(IV), Cr(V), and Cr(III), can interact with DNA [24–26]. Recent work has also shown that Cr(VI) can exert its effects via epigenetic mechanisms. Cr(VI) exposure was shown to increase DNA methylation and silencing of a bacterial reporter gene in human cell lines [27]. In plants, Cr(VI) exposure causes genome-wide DNA hypermethylation [28]. Also, exposure of human lung cancer cell lines (A549) to potassium chromate induces global changes in various histone tail modifications [29]. Furthermore, chromate exposure increased H3K9 dimethylation in the MLH1 promoter, leading to decreased MLH1 expression [29].

## Results

To test if Cr(VI) exposure induces phenotypic variations in the progeny, we exposed male and female *Kr^If-1^* flies to various concentrations of K_2_Cr_2_O_7_, a Cr(VI) compound, and scored for phenotypic variations in F1 flies. The concentration of Cr(VI) used in this study is consistent with previous cell line exposure studies [8]. *Kr^If-1^* is a spontaneous mutation and a gain of function allele of *Krüppel* (*Kr*)—a zinc finger transcription factor required during early embryogenesis [30]. The exact nature of the *Kr^If-1^* allele has not been characterized yet. *Kr^If-1^* results in ectopic expression of *Kr* in the ventral region of the eye imaginal disc, which, in turn, misregulates homeotic genes resulting in eye outgrowths if not repressed by developmental robustness [31, 32]. Using this model system, mutations in Hsp83, *trx* group proteins, and the piRNA pathway induced phenotypic variations in the progeny [4, 6]. We noted that exposure of parents to Cr(VI) exposure led to eye phenotypic variations (Fig. 1b) in the offspring in a dose-dependent manner (Fig. 1c), suggesting that the phenotypic variations were produced in response to the presence of Cr(VI) in the food. These phenotypic variations are similar to those induced by the loss-of-function alleles of *piwi, aub, Hsp83*, and Trithorax-group genes [4, 6]. From here on, Cr(VI) refers to K_2_Cr_2_O_7_. Cr(VI) exposure did not affect the protein levels of Hsp83 and Piwi (Fig. 1d), suggesting that phenotypic variations were due to alterations in the post-translational function of these proteins.

**Figure 2:**
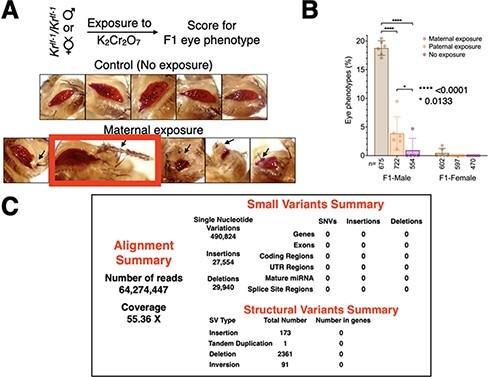
Cr(VI) exposure–dependent developmental defects in the progeny are not due to genetic mutations. (a) Eye phenotypes in the progeny upon exposure of only mothers to 10 μM Cr(VI) for 6 days. (b) Quantitation of phenotypes in the progeny upon either maternal or paternal exposure to 10 μM Cr(VI). Maternal exposure has a significant effect when compared to paternal exposure. (c) Results from bioinformatic analysis of single-fly whole genome sequencing. An image of the fly eye with phenotypic variation used for whole genome sequencing is shown in a highlighted box in A. A summary of small variations and large structural variations in the genome is also shown. Mutations that would directly affect the function of a gene directly did not occur upon exposure to 10 μM Cr(VI)

To further characterize the Cr(VI) exposure effect on F1 development, we exposed either males or females to Cr(VI) and allowed them to mate with the opposite sex flies that were never exposed to Cr(VI). Exposure of females to Cr(VI) produced 3- to 4-fold more F1 phenotypic variations than the exposure of males (Fig. 2a and b). The most extreme phenotypic variation was the growth of an appendage from the ventral region of the eye (Fig. 2a, highlighted box). This result led us to conclude that the exposure of parents, especially mothers, to Cr(VI) can derail cell fate determination in the progeny. To test if Cr(VI) exposure–induced phenotypic variations in F1 are due to novel genetic mutations, we performed single-fly whole genome sequencing to identify, if any, Cr(VI) exposure–induced DNA mutations. For the robustness of the procedure, we performed whole genome sequencing using two independent flies with two different phenotypic variations (Figs 2c, S1, and S2). Data analysis was performed using the BWA Whole Genome Sequencing (v1.0) BaseSpace Workflow from Illumina (see Methods for more details) using the *dm3* genome as the reference. While we noted small variants and large structural variations in the genome in flies with different phenotypes compared to the reference genome, none of these variations were within genes, 5ʹ and 3ʹ UTRs, mature miRNAs, or in splice site regions (Figs 2c, S1, and S2). This result shows that Cr(VI) exposure–dependent phenotypic variations shown in Figs 1 and 2a are not caused by mutations that directly affect the function of a gene; perhaps inherent genetic variations or *de novo* mutations in the non-coding portion of the genome results in altered phenotypes.

**Figure 3:**
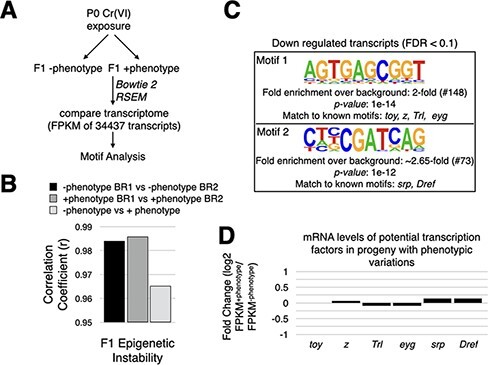
Cr(VI) exposure induces epigenetic instability in F1 somatic tissues. (a) An experimental scheme to identify differentially expressed transcripts. (b) Evidence for epigenetic instability in F1 somatic tissues. We measured epigenetic instability by comparing transcriptomes of indicated fly heads and plotting the correlation coefficient (*r*). (c) Motifs enriched in promoter elements of downregulated genes are shown. Potential transcription factors that bind to these motifs are also shown. The number in parentheses represents the number of differentially expressed genes that possess a match to the respective motif. (d) The mRNA levels of potential transcription factors that can bind to the motifs are shown in C. Fold-change represents log2 (FPKM + phenotype/FPKM − phenotype). FPKM values were obtained from mRNA-seq analysis using RSEM (see Methods). FPKM from two biological repeats were averaged prior to fold-change calculation

Epigenetic instability altering gene transcription can increase phenotypic diversity [33]. We reasoned that if the Cr(VI) exposure–induced phenotypic variations were due to epigenetic instability, we should note a significant difference in the transcriptome of flies with and without phenotypic variations. To this end, we performed mRNA-seq of head tissues of the progeny with and without phenotypic variations, compared to their transcriptomes [fragments per kilobase of transcript per million mapped reads (FPKM) of 34 437 transcripts], and calculated the *Pearson* correlation coefficient (*r*) (Fig. 3a). A decrease in transcriptome correlation (*r*) implies more significant variability in gene expression patterns, in other words, greater epigenetic instability. A comparison of transcriptomes between biological repeats of the same condition yielded an *r* of ∼0.99, suggesting that the two independent biological repeats have similar transcriptomes. On the other hand, transcriptomes of flies with and without phenotypes had a reduced *r* of ∼0.96, suggesting that phenotypic variations are associated with a significant change in the transcriptome (Fig. 3b). The decrease in *r* was driven by differential expression of ∼11% of the transcriptome (3731 transcripts, false discovery rate (FDR) <0.1). Of the 3731 transcripts, 618 (∼16.5%) and 789 (∼21%) exhibited >2-fold upregulation and downregulation, respectively.

To test if a specific set of transcription factors drove the differential expression of genes, we analyzed promoter elements [−400 to +100 of the transcription start site (TSS)] of up- and downregulated genes for enriched motifs using HOMER [34]. About 28% of downregulated genes were enriched for two motifs (Fig. 3c). Motif 1 matches the binding sites of known proteins *twin of eyeless* (*toy*), *zeste* (*z*), *Trithorax-like* (*Trl*), and *eyegone* (*eyg*). *Toy* and *eyg* are paired homeobox transcription factors critical for eye development [35, 36]. On the other hand, *z* and *Trl* belong to the Polycomb group (PcG) recruiters/DNA-binding proteins and are directly involved in regulating chromatin compaction [37]. In an independent study, a reduction in maternal dosage of *z* and *Trl* significantly increased phenotypic variations in the *Kr^If-1^* model system [4]. Motif 2 matches the binding sites of “serpent” (*srp*) and “DNA replication-related element factor” (*Dref*). While *srp* is a vital transcription factor required for the development of mesodermal derivatives [38], *Dref* is needed for compound eye morphogenesis [39]. Downregulation of the transcripts containing these motifs in their respective promoter elements was not due to the misexpression of *toy, z, Trl, eyg*, *srp*, and *Dref* (Fig. 3d). This result suggests that phenotypic variations are not due to a change in the levels of the aforementioned transcription factors; perhaps chromatin accessibility for these transcription factors changes in F1. The difference in chromatin accessibility increases epigenetic instability. In contrast, upregulated genes did not show enrichment of any motif.

**Figure 4:**
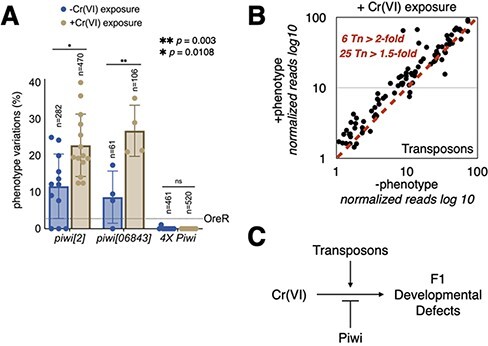
The relationship between Cr(VI) exposure and Piwi. (a) A column plot showing the relationship between the level of Piwi mRNA in the mother’s germline and F1 phenotypic variations. The number of flies assessed for phenotypic variations are shown. (b) A scatter plot showing the levels of transposon mRNAs in fly heads with and without phenotypic variations. Reads were normalized as counts per million mapped reads. (c) An illustration summarizing the findings in A and B

We have previously shown that a decrease in the maternal dosage of Piwi, a piRNA-binding factor, leads to phenotypic variations in F1, much like those seen with Cr(VI) exposure [6]. Piwi is the founding member of the PIWI class of proteins that bind ∼26-nucleotide-long small non-coding RNAs called piRNAs [40–42]. Piwi uses the sequence of the bound piRNAs, targets the anti-sense sequence in the transposon target RNA, and induces epigenetic silencing of transposons [43–54]. A lack of Piwi function leads to the activation of transposons and a sterile phenotype [55]. To explore the relationship between Piwi and Cr(VI) exposure, if any, we exposed mothers with different levels of Piwi mRNA to Cr(VI) and measured phenotypic variations in F1. The presence of only one functional copy of Piwi (*piwi^2^* or *piwi^06843^*) led to an increase in phenotypic variations when compared to *OreR* (wild-type control) (Fig. 4a, blue columns). Cr(VI) exposure led to a further rise in phenotypic variations when compared to unexposed flies suggesting that a Piwi dosage decrease and Cr(VI) exposure have an additive effect on F1 phenotype variations (Fig. 4a, brown columns). However, when we increased the dosage of Piwi to 4, we noted that Cr(VI)-dependent phenotypic variations reduced significantly in both Cr(VI)-unexposed and -exposed conditions. These data provide direct evidence that the Piwi–piRNA pathway acts as an immune system to prevent environmental insult–induced epigenetic changes that can derail developmental robustness. Since overexpression of Piwi was able to reduce Cr(VI) exposure–dependent F1 phenotypic variations and Piwi’s primary function is to silence transposons, we hypothesized that F1 phenotypic variations are due to increased transposon activity. To test this, we measured transposon mRNA levels in F1 heads with and without phenotypic variations using mRNA-seq and analysis by piPipes [56]. We detected phenotype-dependent increases in the steady-state levels of transposons. Specifically, we noted that 6 and 25 transposon families were activated >2- and 1.5-fold, respectively (Fig. 4b). Independent analysis using the RSEM-EBSeq [57, 58] workflow showed that 15 transposon families were activated with a FDR cutoff of 0.1 in a phenotype-dependent manner (Fig. S3). This result strongly suggests that robust transposon silencing in the somatic tissues of the offspring gets derailed when the mother is exposed to Cr(VI).

**Figure 5:**
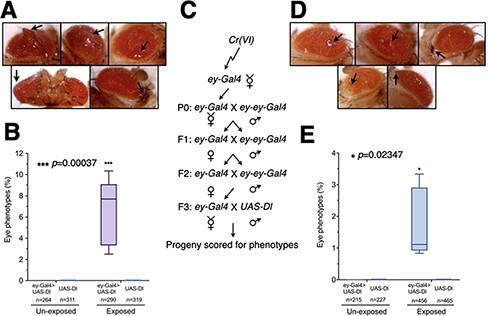
Cr(VI) exposure–dependent transgenerational predisposition to cancer. (a) Drosophila eyes (ey-Gal4 > Delta) showing tumors (black arrows) when exposed to Cr(VI). In addition to tumors, we also noted bristle phenotypes. Follow the black arrows. (b) Quantitation of phenotypes observed in A. The experiment was performed in triplicates. (c) A flowchart showing the crossing scheme used to test if Cr(VI) exposure–induced epigenetic instability is fixed in a population for multiple generations. (d) F4 fly eyes exhibiting tumors and bristle phenotype (black arrows). (e) Quantitation of phenotypes observed in D

Based on the data, we conclude that Cr(VI) exposure causes epigenetic instability in the progeny, affecting cell fate commitment decisions. This raises two questions: (i) are the epigenetic instability and phenotypic variations in F1 specific to the *Kr^If-1^* model system? and (ii) can Cr(VI) exposure–induced F1 epigenetic instability lead to disease states like tumors? We used a Delta (Dl; a Notch ligand) overexpression-based tumorigenesis model to answer these questions. In this model, *Dl* is overexpressed in the eye using *ey-Gal4*. Using this model, mutations in the members of the PcG proteins were shown to induce metastatic tumors in *Drosophila* [59]. So, we tested if epigenetic instability in the progeny caused by Cr(VI) exposure can lead to tumors in *Drosophila*. We exposed male and female *ey-Gal4* to 50 µM Cr(VI) in fly food for 6 days and then transferred them to new fly food with no Cr(VI). The progeny from the cross were then scored for eye tumors. As a control, flies were exposed to fly food with no Cr(VI). We noted that 8–10% of F1 flies developed eye tumors (Fig. 5a and b, follow the black arrows in A and Fig. S4a). In contrast, progeny flies from the control cross did not show tumors (see quantification in Fig. 5b). Next, we tested if tumor development is fixed in the population. Following the crossing scheme shown in Fig. 5c, we mated F3 *ey-Gal4* females with *UAS-Dl* males and scored the progeny for eye tumors. Significantly, even after initial exposure to Cr(VI), F3 females could still induce eye tumors in the F4, albeit less efficiently (Figs 5d and e and S4b). This data show that Cr(VI) exposure–induced predisposition to develop tumors in the progeny gets fixed in the population for at least three generations. These results show for the first time that Cr(VI) exposure can lead to tumors in the progeny who have not been exposed to Cr(VI) and that the epigenetic instability induced by Cr(VI) exposure can be fixed in a population for at least three generations.

## Discussion

This work puts forward the following four key concepts: (i) environmental pollution can derail developmental robustness leading to long-lasting detrimental effects on the progeny, (ii) Cr(VI) exposure not only affects the exposed individual but also derails the development of the progeny, (iii) piRNA pathway can act as an organismal immune system to protect evolutionarily favored phenotypes from environmental insults, and (iv) epigenetic instability induced by Cr(VI) exposure can get fixed in a population for at least three generations leading to a predisposition to develop tumors.

**Figure 6:**
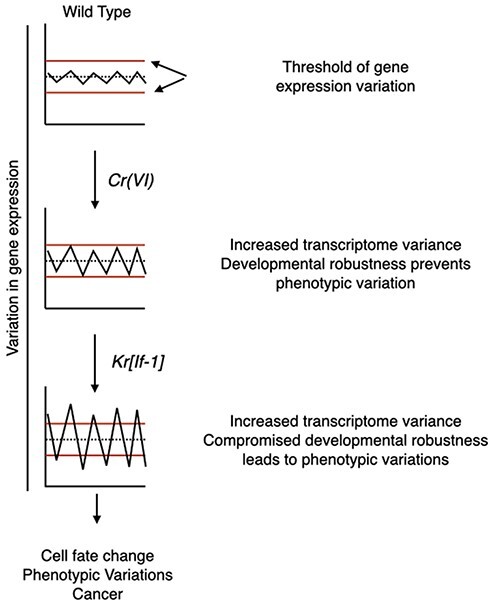
a model showing the effect of Cr(VI) exposure on the robustness of epigenetic inheritance in the offspring

The memory of development from a zygote to an adult is transmitted from one generation to the next through a process called epigenetic inheritance. This germline-inherited epigenetic memory is a collaboration of several cellular mechanisms that include transcription factors, DNA methylation, histone modifications, and non-coding RNAs [60]. Faithful epigenetic inheritance is, in turn, essential for developmental robustness. While Cr(VI) is a known mutagen, it is slowly becoming apparent that epigenetic regulation forms a basis for its carcinogenicity [61]. However, it was unknown whether Cr(VI) exposure–induced epigenetic dysregulation could be inherited across generations. In this work, we show for the first time that parents’ Cr(VI) exposure affects F1 development without impacting the underlying genetic sequence and that these effects could be inherited in later generations. We propose that Cr(VI) exposure induces F1 phenotypic variations and tumors via two steps: (i) Cr(VI) exposure would first derail epigenetic inheritance leading to the predisposition of the progeny, and (ii) misexpression of factors that change transcription patterns in predisposed the progeny would then increase epigenetic instability leading to phenotypic variations and tumors (Fig. 6). In support of this two-step model, we show that *Kr^If-1^* and overexpression of *Dl* in the absence of Cr(VI) exposure fail to generate phenotypic variations and tumors, respectively (Figs 1c and 5b).

How does impaired epigenetic inheritance induce epigenetic instability? Mechanistically, the impaired epigenetic inheritance would allow or prevent *Kr*, transcription factors functioning downstream of *Dl*, and other transcription factors (Fig. 1c and d) from gaining access to chromatin sites that would otherwise be inaccessible in the progeny of non-exposed parents. In support of this, we show that several downregulated genes in flies with phenotypic variations possess binding sites for trithorax-like gene (*Trl*)—a trxG gene (Fig. 1c) [62]. Trl, also known as the GAGA factor, is a transcriptional activator for several homeotic genes; however, it can also act as a transcriptional repressor. Modified chromatin accessibility to Kr, the Notch signaling pathway, and Trl would change the transcriptome and induce epigenetic instability and phenotypic variations. How Cr(VI) exposure derails robust epigenetic inheritance is not clear yet. Further mechanistic work into parental germline response to Cr(VI) exposure is needed to answer this. One possibility is that Cr(VI) exposure affects the function of the PcG and trithorax group (trxG) of proteins; these proteins have a critical role in epigenetic inheritance and the expression of the homeotic selector (HOX) genes [37].

It is interesting to note that increasing the dosage of Piwi could reduce Cr(VI) exposure–dependent phenotypic variations. This is in line with the earlier finding that overexpression of Piwi reduces Hsp83 mutation–induced phenotypic variation [6]. The exact mechanism by which Piwi achieves this is not very clear. Piwi may prevent transposon activation–induced *de novo* mutations [63–65] or the effect of transposon activation on neighboring gene activation [5]. We believe that any impact of transposon activation would have to happen in the non-coding portion of the genome, as we did not notice any significant structural changes in the coding part of the genome (Fig. 2c). How does Cr(VI) exposure lead to the activation of transposons? It is tempting to speculate that Cr(VI) exposure would affect piRNA biogenesis [40] in the germline of the exposed mother or the function of endogenous short-interfering RNA (siRNA) in the heads [66]. Piwi could also reduce Cr(VI) exposure–dependent phenotype variations in a transposon-silencing-independent manner. A potential clue comes from recent work showing that Piwi physically interacts with Polycomb group complex PRC2 subunits Su(z)12 and Esc and sequesters PRC2 in the nucleoplasm [67]. This, in turn, would attenuate the Polycomb group function. Perhaps Piwi’s attenuating PRC2 function would normalize the transcriptome change induced by the modified chromatin accessibility of Trl—a trxG protein [62]. It is known that PcG and trxG have antagonistic roles in regulating gene expression.

It is clear from our work that Cr(VI)-induced effects are transmitted to the next generation predominantly via the oocyte, as Cr(VI)-exposed females produced significantly more flies with the phenotype variations than the exposed males (Fig. 2b). How Cr(VI) exposure affects the oocyte remains to be characterized. Recent research has shown that the covalent histone mark H3K27me3 can be transmitted from the oocyte to the offspring, and lack of the same leads to aberrant activation of lineage-specific genes during the activation of the zygotic genome [68]. The inheritance of covalent histone marks from the oocyte to the offspring is conserved in mice [69, 70]. One possibility is that Cr(VI) exposure affects the inheritance of covalent histone marks from the oocyte to the offspring. Characterization of the epigenome of the oocyte upon Cr(VI) exposure will provide further insights into the same. Small non-coding RNAs, especially piRNAs, have been demonstrated to mediate the transgenerational inheritance of acquired traits [71] or modulate the expression of transgenes across generations [72]. It will be interesting to see if Cr(VI) exposure affects piRNA levels or the signature of the piRNAs in the exposed mother’s germline.

This work lays the foundation to use *Drosophila* as a model system to unravel the epigenetic mechanism by which Cr(VI) exposure affects organismal development and identify novel genetic targets that can potentially suppress Cr(VI) exposure–induced F1 defects. Furthermore, this model system could screen for environmental pollutants that can act as teratogens and provide a genetic basis for the same. Considering this study’s findings, epidemiological studies on the progeny of Cr(VI)-exposed parents are warranted.

## Methods

### Fly Stocks, RNAi, and Maintenance

All *Drosophila* stocks were maintained at 25**°**C and fed on corn meal–based media (Nutri-fly, Bloomington formulation by Genesse Scientific). Fly food was prepared as per product directions. For preparing Cr(VI)-containing food, the required amount of aqueous solution of K_2_Cr_2_O_7_ was added to 10 ml food after pouring it into vials at 65–70°C. Food was stirred with a sterile spatula to mix Cr(VI) properly. *b^1^,Kr^If-1^* (No. 4194), w*; P{UAS-Dl.J}3 (No. 26695), and w*; P{GAL4-ey.H}4-8/CyO (No. 5535) were obtained from the Bloomington stock center (stock numbers are indicated in parentheses).

### Cr(VI) Exposure

In the direct exposure experiment (Fig. 1b and c), 4–5 virgin female flies and three 2- to 3-day-old male flies were added to vials with food mixed with various concentrations of K_2_Cr_2_O_7_ (1, 2.5, 5, 7.5, 10, 50, and 100 µM) and the progeny was scored for phenotypes. The percentage of progeny exhibiting developmental defects reached a plateau at 10 µM with 10 and 50 µM exposures affecting a similar percentage of F1 flies. Flies exposed to 100 µM K_2_Cr_2_O_7_ died within 6 days. Flies were retained on K_2_Cr_2_O_7_-containing food for the entirety of the experiment. Since 10 µM K_2_Cr_2_O_7_ had the most significant effect without causing lethality, we used 10 µM for all the experiments in this work except for sex-specific exposure (Fig. 2a and b), in which ∼10 to 15 virgin female and male flies were first exposed to 10 µM K_2_Cr_2_O_7_-containing food for 6 days before mating with the opposite sex that was never exposed to K_2_Cr_2_O_7_. Mating was performed on food without K_2_Cr_2_O_7_. The progeny from this cross was then scored for phenotypes. A similar strategy was followed for studying the relationship between Piwi and Cr(VI) exposure, except that females carrying various *piwi* alleles, as shown in Fig. 4a, were exposed to 10 µM K_2_Cr_2_O_7_ Cr(VI) and then mated with 2- to 3-day-old male *Kr^If-1^* flies. Both direct exposure and sex-specific exposure experiments were repeated at least three times with at least three independent crosses. Parents were transferred to fresh food every 3–4 days.

### Ovary Lysate Preparation and Immunoblotting

Ovary lysate preparation and Western blotting were performed as described earlier. The lysate was mixed with an equal volume of microliters of 2× Laemmli Sample Buffer, boiled at 95°C for 3 min, and then processed for Western blotting using the manufacturer’s instructions (Bio-Rad). α-Hsp83 (Cell Signaling Technology, #4874), α-Piwi, and α-Act5c (Cell Signaling Technology, #4967) antibodies were used at 1:1000, 1:5000, and 1:1000, respectively. Piwi antibody was a generous gift from the Haifan Lin lab.

### Statistical Analysis

Statistical analyses were performed in GraphPad Prism 9.0 using Analysis of variance (Figs 1c and 2b) and a *t*-test (Figs 4a and 5b and e). *p* ≤ 0.05 was considered significant in all statistical tests.

### Genomic DNA Preparation and Whole Genome Sequencing

Using ethanol extraction, genomic DNA from a single fly (with phenotype) was prepared. DNA (∼100 ng) was sheared to an average of 160 bp using a Covaris S220 sonicator. Sequencing libraries were prepared from 20 ng of sheared DNA utilizing the Illumina Truseq RNA v2 kit, starting the end repair step following the manufacturer’s protocol. Paired-end 125 bp sequencing was performed on Illumina HiSeq2500.

### mRNA-Seq

Total RNA was isolated from 10 heads using TRIzol (Thermo Fisher Scientific), and mRNA sequencing was performed as described elsewhere [73].

### Bioinformatics

#### DNA Sequencing Analysis

Whole genome sequencing data analysis was performed using the BWA Whole Genome Sequencing (v1.0) BaseSpace Workflow (Illumina). Within this workflow, DNA sequencing reads were aligned to the *dm3* release of the *Drosophila* genome using BWA [74] and processed using SAMtools [75, 76]. Genome Analysis Toolkit [77] was used to call variants.

#### mRNA Sequencing Analysis

RNA sequencing data analysis was performed as described earlier [73]. Briefly, raw sequencing reads were aligned to Refseq mRNA (dm6) genes using Bowtie 2 [78] and quantified using RSEM [57]. The Pearson correlation coefficient (*r*) shown in Fig. 3b was calculated by comparing the FPKMs of 34 437 transcripts. FPKMs from two independent biological repeats were averaged before calculating *r*. Differential expression analysis was performed using EBSeq [58]. Differentially expressed transcripts with an FDR <0.1 were deemed statistically significant.

#### Motif Analysis

To identify enriched motifs in the promoter elements of differentially expressed genes, we used HOMER’s *findMotifs.pl* script [34]. Motifs of 8 and 10 nucleotides enriched in −400 to +100 relative to the TSS were identified.

#### Transposon mRNA Levels

Transposon mRNA levels shown in Fig. 4b were calculated using the piPipes RNA-seq pipeline [56]. The RSEM-EBSeq workflow was used to calculate transposon mRNA levels in flies with and without phenotype variations as described elsewhere [73].

## Supplementary Material

dvac030_Supp

## Data Availability

Sequencing data from this study can be accessed using Gene Expression Omnibus (GEO) accession number GSE111834.
