# Supplementary material for: Hexavalent chromium–induced epigenetic instability and transposon activation lead to phenotypic variations and tumors in Drosophila
Source: Environ Epigenet. 2022 Dec 28;9(1):dvac030. doi: 10.1093/eep/dvac030 (PMC9892686; doi:10.1093/eep/dvac030)
Supplement: dvac030_Supp [file dvac030_supp.zip › suppl_data/Supplementary Info_Revised.pdf]

Supplementary Figure Legends

Figure S1: Summary of single fly whole genome sequencing. Please note that this is summary of the same fly shown in figure 2C. Read 1 and Read 2 represent the two reads of paired-end sequencing.

Figure S2: same as figure S1 except that a different fly with a different phenotypic variation was used.

Figure S3: A column plot showing the steady state levels of transposon mRNAs in the heads of flies with and without phenotypes.

Figure S4: Enlarged images of a few eyes exhibiting cancer growths shown in figures 5A and 5D.

Single fly whole genome sequencing
Fly #1
Sex: Male

Alignment Summary

Number of reads
64,274,447

Coverage
55.36X

Percent Duplicate Paired Reads
53.2%

Fragment Length Median
163bp

Fragment Length Standard Deviation
62bp

Read Statistics

	% Aligned	% Q30	Mismatch Rate
Read 1	51.11%	88.79%	0.87%
Read 2	47.32%	90.00%	0.91%

Figure S1

Single fly whole genome sequencing
Fly #2
Sex: Male

Alignment Summary

Number of reads
45,448,843

Coverage
29.23X

Percent Duplicate Paired Reads
54.8%

Fragment Length Median
165bp

Fragment Length Standard Deviation
77bp

Small Variants Summary

Single Nucleotide Variations
279,204

Insertions
10,690

Deletions
11,299

Variations by Sequence Context

	SNVs	Insertions	Deletions
Genes	0	0	0
Exons	0	0	0
Coding Regions	0	0	0
UTR Regions	0	0	0
Mature miRNA	0	0	0
Splice Site Regions	0	0	0

Structural Variants Summary

Insertions
160

Deletions
1,818

Inversion
186

Tandem Duplication
2

Variations in Genes
0

Read Statistics

	% Aligned	% Q30	Mismatch Rate
Read 1	42.88%	86.97%	1.05%
Read 2	36.62%	86.62%	1.16%

Figure S2

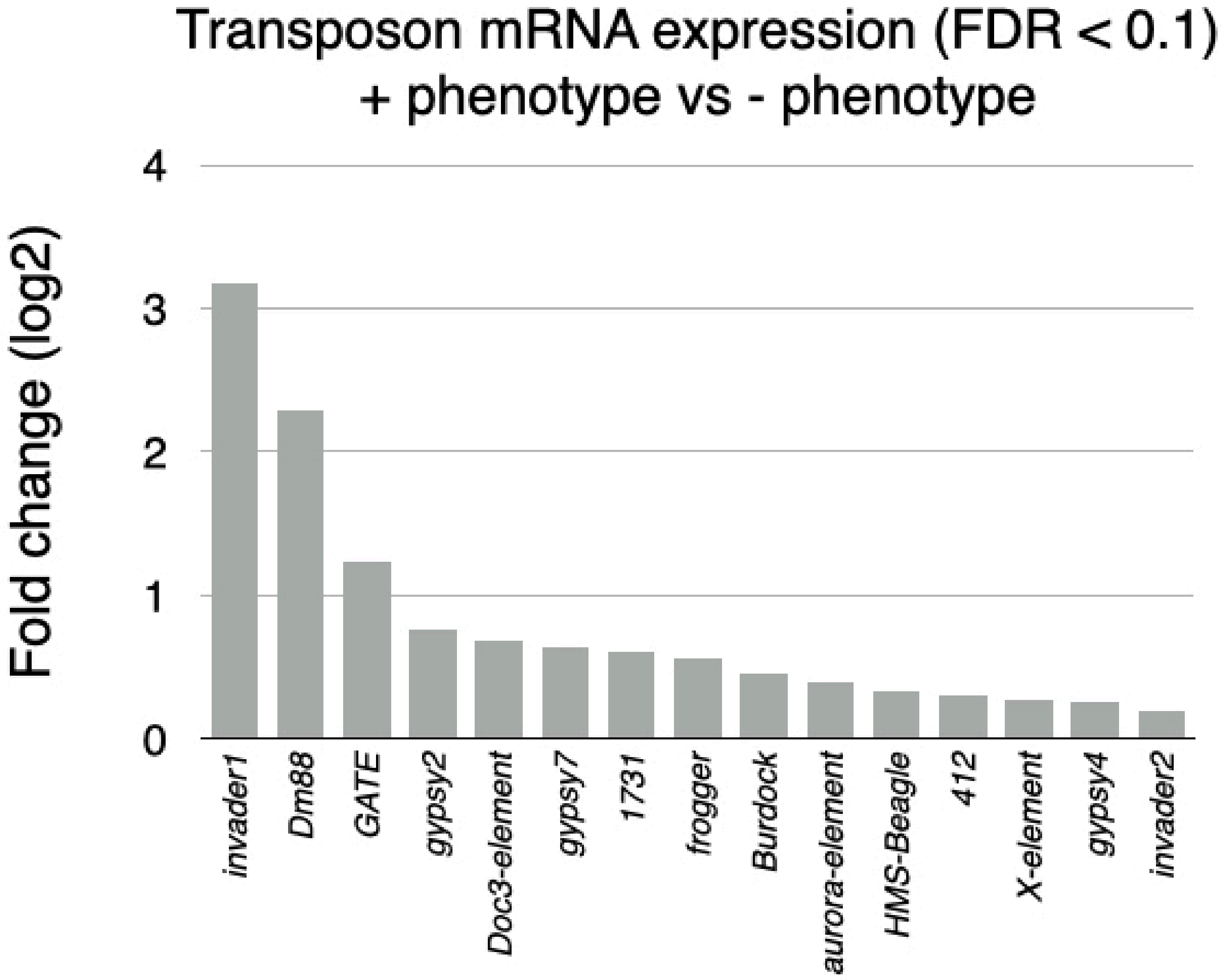

Transposon mRNA expression (FDR < 0.1)
+ phenotype vs - phenotype
Fold change (log2)
4
3
2
1
0
invader1
Dm88
GATE
gypsy2
Doc3-element
gypsy7
1731
frogger
Burdock
aurora-element
HMS-Beagle
412
X-element
gypsy4
invader2

Figure S3

A

F1 flies from figure 5A

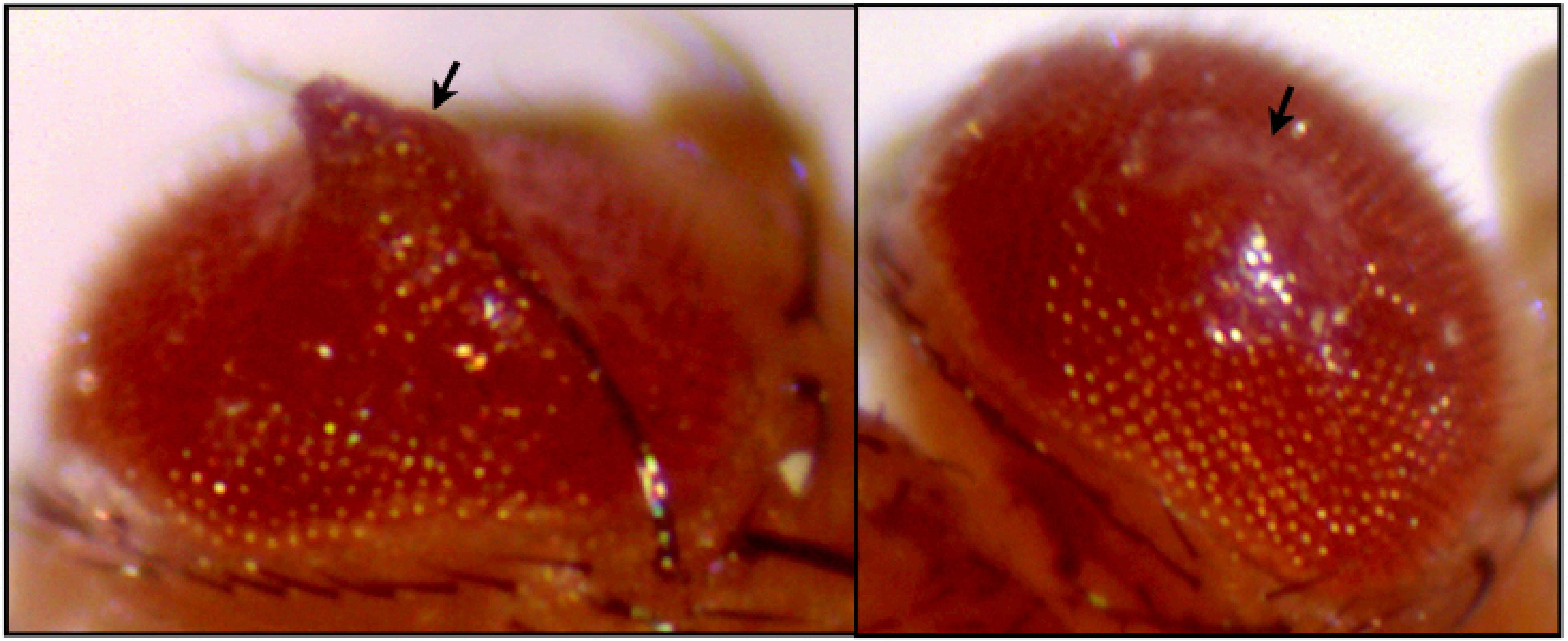

B

F4 flies from figure 5D

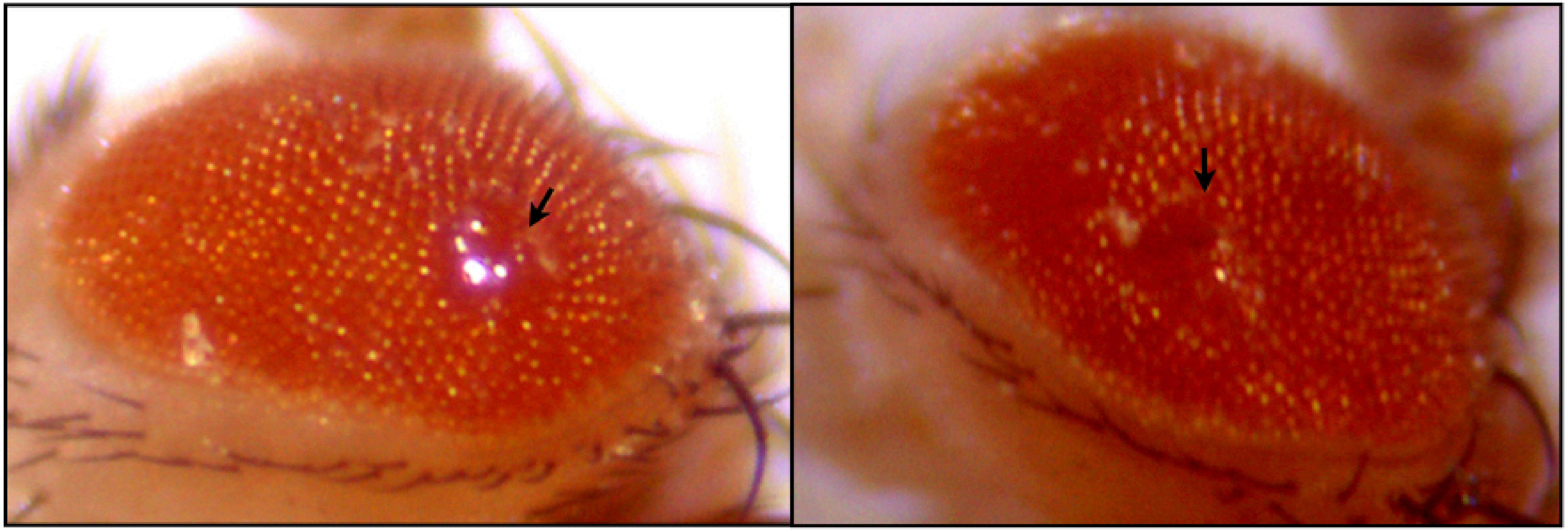

Figure S4
